# Mesoporous polydopamine delivering 8-gingerol for the target and synergistic treatment to the spinal cord injury

**DOI:** 10.1186/s12951-023-01896-1

**Published:** 2023-06-14

**Authors:** Jinpei Yang, Meng Wang, Shuai Zheng, Ruodong Huang, Ganjun Wen, Pan Zhou, Wenbo Wang, Shihao Zhou, Xinlin Jiang, Shuangjiang Liu, Zhizhong Li, Dong Ma, Genlong Jiao

**Affiliations:** 1 Department of Orthopaedics, the First Affiliated Hospital of Jinan University, Jinan University, 613 Huangpu Avenue West Road, Guangzhou, 510630 Guangdong China; 2 Department of Orthopaedics, Huizhou Third People’s Hospital, Guangzhou Medical University, Huizhou, 516002 Guangdong China; 3 The Sixth Affiliated Hospital of Jinan University, Jinan University, Dongguan, 523573 Guangdong China; 4 Key Laboratory of Biomaterials of Guangdong Higher Education Institutes, Engineering Technology Research Center of Drug Carrier of Guangdong, Department of Biomedical Engineering, Jinan University Guangzhou, Guangzhou, 510632 China; 5 MOE Key Laboratory of Tumor Molecular Biology, Jinan University, Guangzhou, 510632 China; 6 The Fifth Affiliated Hospital of Jinan University, Jinan University, Heyuan, 51700 Guangdong China

**Keywords:** Mesoporous polydopamine, Nano-delivery platform, Spinal cord injury, Ferroptosis, 8-gingerol, Cerebrospinal fluid

## Abstract

In the treatment of spinal cord injury (SCI), the complex process of secondary injury is mainly responsible for preventing SCI repair or even exacerbating the injury. In this experiment, we constructed the 8-gingerol (8G)-loaded mesoporous polydopamine (M-PDA), M@8G, as the in vivo targeting nano-delivery platform, and investigated the therapeutic effects of M@8G in secondary SCI and its related mechanisms. The results indicated that M@8G could penetrate the blood-spinal cord barrier to enrich the spinal cord injury site. Mechanism research has shown that all of the M-PDA,8G and M@8G displayed the anti-lipid peroxidation effect, and then M@8G can inhibit the secondary SCI by suppressing the ferroptosis and inflammation. In vivo assays showed that M@8G significantly diminished the local injury area, reduced axonal and myelin loss, thus improving the neurological and motor recovery in rats. Based on the analysis of cerebrospinal fluid samples from patients, ferroptosis occurred locally in SCI and continued to progress in patients during the acute phase of SCI as well as the stage after their clinical surgery. This study showcases effective treatment of SCI through the aggregation and synergistic effect of M@8G in focal areas, providing a safe and promising strategy for the clinical treatment of SCI.

**Supplementary Information:**

The online version contains supplementary material available at 10.1186/s12951-023-01896-1.

## Introduction

Spinal cord injury (SCI) is often caused by traumatic factors and includes both primary and secondary stages. The primary phase often results in the disruption of nerve fiber bundles, local capillaries and the Blood-Spinal Cord Barrier (BSCB). As the primary phase cannot be altered, the secondary phase, which occurs thereafter with secondary injury, is the main phase of current research. Secondary injury often involves inflammation, cellular autophagy, necrosis, apoptosis and ferroptosis, which leads to persistent neuronal cell death, axonal demyelination and scarring, ultimately causing permanent functional impairment [1, 2]. Currently, clinical management of secondary SCI is still confined to the use of high doses of methylprednisolone, surgical interventions for stabilization and spinal cord decompression, and rehabilitation nursing care, but their treatment outcomes are unsatisfactory [3–5].

Extensive research has shown that one of the most important mechanisms in secondary SCI is the excessive production of post-traumatic reactive oxygen species (ROS) and its consequent oxidative damage induced by oxygen radicals [6–8], including DNA, lipids and proteins [9, 10]. 8-Gingerol (8G) is a phenolic compound, one of the main functional substances of ginger with strong anti-oxidative stress, anti-inflammatory, immunosuppressive and anti-tumor efficacy [10–13]. In addition, 8G can inhibit cellular autophagy and apoptosis by reducing the production of ROS in excess [14]. At present, no studies are available for evaluating the therapeutic effects of 8G on SCI. To make a breakthrough in this field, we have carried out experiments by using 8G to assess its efficacy of secondary SCI treatment. Studies have shown that ROS-induced lipid peroxidation (LPO) plays a key role in cell death, including apoptosis, autophagy and ferroptosis [9]. Given the excellent anti-ROS effect of 8G, we speculate that 8G may be associated with ferroptosis. Ferroptosis is a non-apoptotic form of programmed cell death characterized by intracellular iron overload and LPO [15].Some research findings have shown that ferroptosis is one of the important mechanisms involved in secondary SCI, and that ferroptotic inhibitors can effectively suppress SCI [16, 17]. On this basis, we investigated the therapeutic and inhibitory effects of 8G on ferroptosis in secondary SCI in this experiment. However, 8G is poorly soluble, easily inactivated in vivo and rapidly metabolized, resulting in low bioavailability. Therefore, the key to the treatment of secondary SCI with 8G is constructing a drug delivery vehicle that can efficiently load 8G and target delivery to the site of spinal cord injury, so as to enrich8G at the site of spinal cord injury.

Drugs with potential neuroprotective effects often have reduced clinical efficacy due to rapid metabolism or failure to cross the blood-spinal cord barrier. Thanks to the development and application of biomaterials, nanomaterials are bringing a new vision for the treatment and research of SCI [18, 19]. Specifically, polydopamine (PDA) can form stable structures with drug molecules by using the surface catechol structure to form a large number of π-π conjugated hydrogen bonds with drug molecules, making PDA nano-systems loaded with drug molecules have the potential to become a platform for in vivo drug delivery [20, 21]. Nevertheless, conventional PDA nanoparticles are generally non-porous and therefore show limited drug loading capacity [22]. Furthermore, under complex physiological conditions, drugs absorbed on the surface of nanoparticles can be easily dislodged [23]. For that reason, mesoporous PDA nanoparticles, which can stably load large amounts of drugs in their pores, are more ideal carriers [24, 25]. Depending on the porosity, MPDA can provide efficient drug encapsulation [23]. Additionally, PDA itself has chemical, physical and biological properties similar to natural melanin in terms of antioxidation, photoprotection, metal chelation and energy dissipation [26–29]. These promising bio-inspired properties give PDA a wide range of applications in bio-imaging, drug delivery, smart hydrogels and photothermal therapy [30–34]. In particular, the outstanding properties of PDA in the rapid scavenging of various free radicals from cells and tissues make it an excellent performer in diverse antioxidant biomedical applications [35–37].

**Fig. 1:**
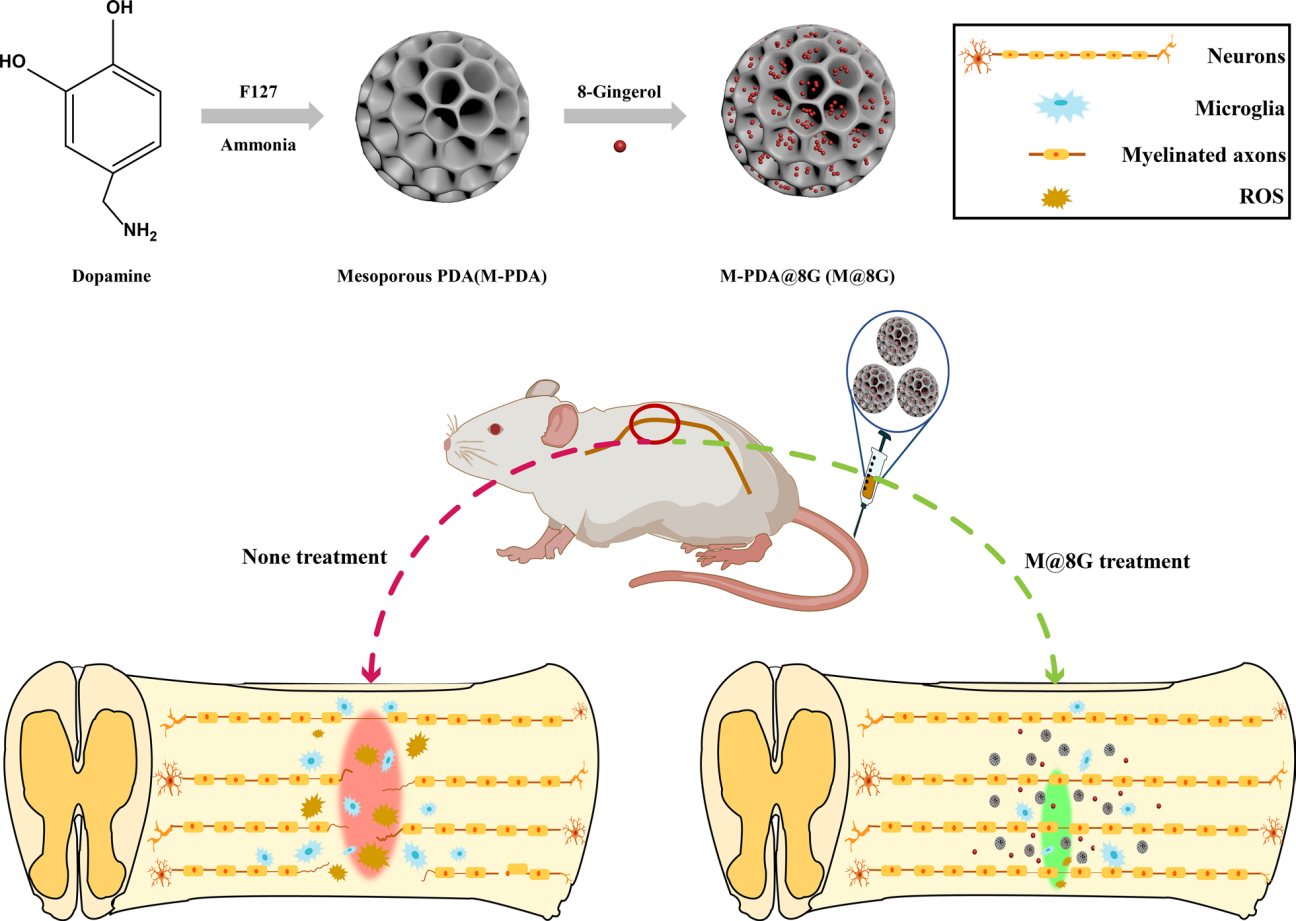
Schematic diagram of the preparation and synergistic treatment of M-PDA@8G for secondary spinal cord injury

In this experiment, a clinically appropriate mesoporous polydopamine nano-delivery platform was designed where dopamine is oxidized by catechol to form quinones, which can subsequently bind to adjacent catechol and/or quinones to produce a thin layer of polydopamine (PDA) on the solid surface under weakly basic conditions (pH 8-8.5), followed by efficient drug loading by encapsulating 8G in its surface mesopores [38, 39]. The nano-delivery platform circulates in vivo with body fluids, and in an altered microenvironment of the spinal cord injury site, it aggregates at the injury site and depolymerizes under acidic conditions to release 8G for therapeutic purposes. Moreover, PDA has the ability to scavenge oxygen radicals, accordingly, it realizes the synergy effect with 8G to treat SCI and improves the microenvironment of the injury site (Fig. 1).

## Materials and methods

### Materials

Dopamine hydrochloride and 1, 3, 5-trimethylbenzene were purchased from Shanghai McLean. Pluronic F-127 was purchased from Sigma-Aldrich. Methanol, ethanol, and dimethyl sulfoxide (DMSO) were prepared by Shanghai Aladdin. Ammonia was purchased from Guangzhou Chemical Reagent Factory (25%). Glacial acetic acid was provided by Guangzhou Chemical Reagent Factory (AR). DMEM medium, Fetal bovine serum (FBS), trypsin, and penicillin-streptomycin double antibody were purchased from Gibco, USA. Cck-8 kit was purchased from Meilun Biotechnology. RSL3, Ferrostatin-1, 8G was purchased from Selleckchem, USA. C11 BODIPY 581/591 was purchased from GLPBIO, USA. MDA kit, ROS kit was purchased from Beijing Solebo. SOD kit was purchased and built in Nanjing. Cy5.5 NHS ester was obtained from Abcam. Cell membrane Green Fluorescence staining Kit (DiO) and DAPI staining solution were purchased from Shanghai Biyuntian Biotechnology Co., Ltd.

### Material preparation and characterization

####  Preparation of M-PDA

Mesoporous polydopamine (M-PDA) nanoparticles were prepared in one step by the template method: 1.5 g of dopamine hydrochloride and 1 g of Pluronic F127 were dissolved in a mixture of 100 mL of ethanol and water (v_ethanol_/v_water_=1: 1). After sonication, 1.6 mL of 1,3,5-trimethylbenzene was added to the mixture and sonicated until uniformly dispersed in the mixture. Subsequently, 3.75 mL of ammonia was slowly added dropwise to the mixture and the reaction was continued under vigorous stirring conditions at room temperature for 2 h. M-PDA nanoparticles were then obtained [40, 41].

#### Preparation of M-PDA@8-Gingerol

1 mg of 8-Gingerol was dissolved in 1 mL of a mixture of water and dimethyl sulfoxide (v_water_: v_dimethyl sulfoxide_=7: 3), and then sonicated until completely dissolved; 2 mg of M-PDA was added to the above mixture, and after 12 h of reaction, the precipitate was collected by centrifugation (12,000 rpm, 20 min) and then washed with water several times until the supernatant was colorless and transparent to obtain M-PDA@8- Gingerol nanoparticles [42].

#### Physicochemical characterization of M@8G

(1) M-PDA and M@8G nanoparticles were dispersed in deionized water, and their hydration particle size and Zeta potential were evaluated by nano laser particle size analyzer. M@8G was added to a flask and dispersed in PBS (pH = 5.5) containing 0.05% Tween 80. The flask was placed on a shaker (100 rpm) at 37 °C for in vitro drug release studies. At predetermined time points (0.5, 1, 2, 4, 8, 12, 24 h), equal amounts of solution (1 mL) were withdrawn and replaced with equal amounts of fresh solution. The samples were centrifuged at 10,000 rpm for 10 min to exclude nanoparticles. The supernatant was filtered through a 0.22 μm filter and analysed by HPLC to determine the released 8G.Their UV absorption spectra were obtained by high performance liquid chromatography at a flow rate of 1.0 mL/min. Mobile phase: V_methanol_: V_water_: V_glacial acetic acid_ =35: 64: 1; the detection wavelength was 280 nm. Injection volume: 20 µL; temperature: 30℃. After freeze-drying, the samples were pressed into thin slices using potassium bromide tablet method, and then the infrared spectra of each sample were obtained by Fourier transform infrared spectrometer.

(2) Surface morphology characterization of microspheres: M-PDA and M@8G nanoparticles were dropped onto a 200-mesh copper net, respectively, and TEM images were taken by transmission electron microscope. The surface mor-phology of the prepared nanoparticles was observed using field emission scann-ing electron microscope (SEM) at 3 kV after different sample solutions were dropped onto a copper platform and sprayed with gold.

### Cellular experiments

#### Cell culture

Mouse Hippocampal Neurons (HT22) were cultured in DMEM medium containing 10% Fetal Bovine Serum (FBS) and 1% Penicillin/Streptomycin. The cells were incubated in a cell culture incubator at 37 °C with 5% CO_2_.

#### Induction of ferroptosis in HT22 cells

HT22 cells were inoculated into 96-well plates (1 × 10^4^ cells/per well) in a cell culture incubator (37 °C, 5% CO_2_) for 12 h until the cells were plastered, and then treated with 0.0625 µM, 0.125 µM, 0.25 µM, 0.5 µM, 1 µM, 2 µM, 4 µM and 8 µM RSL3 for 6 and 24 h, respectively, and their relative cell viability at the corresponding time point was determined by the Counting Kit-8 (CCK-8) assay to investigate the effect of RSL3 on the induction of ferroptosis in HT22 cells.

####  Cytotoxicity of M-PDA, 8G, M@8G, Fer-1

1 × 10^4^ cells per well were inoculated into 96-well plates and incubated in a cell culture incubator (37 °C, 5% CO_2_) for 12 h. After the cells were plastered, DMEM medium was configured with different concentrations of M-PDA (1.25, 2.5, 5, 10, 20, 50, 100, 200 µg/mL), 8G (1.25, 2.5, 5, 10, 20, 50, 100, 200 µg/mL, 100 µM), M@8G (1.25, 2.5, 5, 10, 20, 50, 100, 200 µg/mL), Fer-1 (1 µM) material solutions, and the solutions were added to the corresponding wells and incubated in a cell incubator for 24 h. The liquids were subsequently washed twice with PBS and replaced with 10% CCK8 working solution and co-incubated over the corresponding timescale, and their OD at 450 nm was measured by enzyme marker to calculate cell viability.

#### Anti-Lipid Peroxidation capacity of M-PDA, 8G, M@8G, Fer-1

HT22 cells were inoculated into 24-well plates (1.5 × 10^5^ cells/well) and incubated in a cell culture incubator (37 °C, 5% CO_2_) until the cells were plastered, then RSL3 (1 µM) was first prepared in DMEM medium and added to all wells except the CON group to induce ferroptosis of the cells for 6 h. The RSL3 solution in the well plates was then discarded, and DMEM medium was used to prepare M- PDA (100 µg/mL), 8G (100 µM), M@8G (100 µg/mL), Fer-1 (1 µM), and then added to the corresponding wells and treated for 6 h. The mixture was discarded, washed twice with PBS, BODIPY-C11 probe (2 µM) was added, incubated for 30 min in a cell incubator (37 °C, 5% CO_2_) protected from light, and then the cells were digested by trypsin digestion and collected for flow analysis [43].

#### M@8G cell co-localization

The ability of HT22 cells to community M@8G was assessed using confocal laser scanning microscopy (CLSM). HT22 cells were inoculated in culture dishes (300,000 cells/well). After the cells were plastered, the original medium was replaced with Cy5.5-labelled M@8G (100 µg/mL) medium and incubation was continued for 1 h, 2 h, 4 and 6 h. The cells were then washed 3 times with PBS and sequentially stained with Cell Plasma Membrane Staining Kit with DiO (Green Fluorescence) for 15 min, fixed with 4% paraformaldehyde for 30 min, and stained with DAPI for 5 min. The culture dishes were then placed under CLSM for fluorescence photography.

#### Intracellular (ROS), (SOD), (MDA) assay

HT22 cells were inoculated into 12-well plates (2.5 × 10^5^ cells/well) and incubated overnight in a cell culture incubator (37 °C, 5% CO_2_), and after the cells were plastered, RSL3 (1 µM) was first prepared in DMEM medium and added to all wells except the CON group to induce ferroptosis of the cells for 6 h. Then the RSL3 solution in the well plates was discarded, and DMEM medium was used to prepare M-PDA (100 µg/mL), 8G (100 µM), M@8G (100 µg/mL) and Fer-1 (1 µM), and the cells were added to the corresponding wells and treated for 6 h.

ROS: The mixture was discarded and DCFH-DA was used as a probe molecule to detect intracellular ROS levels. DCFH-DA diluted 1: 1000 in serum-free culture medium was added and incubated for 20 min in a cell incubator (37 °C, 5% CO_2_), protected from light, and intracellular ROS levels were quantified using a multifunctional enzyme marker (excitation and emission wavelengths of 488 and 525 nm, respectively).

SOD: The mixture was discarded. The cells were digested by trypsin, collected in an EP tube and centrifuged (1000 rpm,10 min); the supernatant was discarded; the precipitated cells were kept; 1 mL PBS was added to resuspend the cells; the cells were centrifuged (1000 rpm,10 min); the precipitated cells were collected; 250 µL PBS was added and the cells were blown gently for dispersion; a cell crusher (power 300 W. Ice water bath, sonication every 3–5 s, four times at 30 s intervals) was used to break up the cells. The cells were grouped according to the kit instructions, mixed with different components and incubated for 20 min in a cell incubator (37 °C, 5% CO_2_) protected from light, and the OD value at 450 nm was measured using a multifunctional enzyme marker.

MDA: The mixture was discarded. The cells were digested by trypsin, collected in an EP tube, and centrifuged (1000 rpm, 10 min); the supernatant was discarded; the precipitated cells were kept; the cells were resuspended by adding 250 µL MDA extract; the cells were broken up with a cell crusher (power 20%, ice water bath, sonication 3 s, at 10 s intervals, repeated 30 times) and centrifuged at 8000 g for 10 min at 4 °C. The supernatant was removed and placed on ice for testing. The mixture was kept in an oil bath at 100 °C for 100 min (tightly covered to prevent moisture loss), cooled in an ice bath, centrifuged at 10,000 g for 10 min at room temperature, and 200 µL of supernatant was aspirated into a 96-well plate to determine the absorbance of each sample at 450 nm, 532 and 600 nm. The absorbance of each sample was measured at 450 nm, 532 and 600 nm.

### Animal experiments

#### Establishment of SCI rats model

Animal experiments were approved by the Experimental Animal Ethics Committee of Jinan University (approval number: 20211014-04). The animals were maintained under controlled ambient temperature (25 ± 1 °C) and humidity (55 ± 5%) in artificial lighting under a 12-h light/12-h dark cycle. 10-week-old female Sprague-Dawley rats (220–250 g) underwent severe contusive spinal cord injury. Briefly, SCI rats were anaesthetized with sodium pentobarbital (35 mg/kg, ip) and underwent T10 laminectomy. The dorsum of the T10 spinal cord segment was struck (displacement 1.4 mm for 0.5 s) with a Louisville Injury System Apparatus impactor (Lisa, Louisville, USA). Postoperatively, gentamicin (10 mg/kg, ih) was given to SCI rats for 3 days. Their bladder was massaged twice daily to assist urination until the animal urinated freely. As female rats have a lower mortality rate than male rats in establishing the model, they recover better than males in postoperative function and have fewer postoperative urinary complications than males, making postoperative care more convenient. Therefore, female rats were chosen to establish an animal model of spinal cord injury.

#### In vivo targeting capability of M-PDA

M-PDA was loaded with CY5.5 (1 mg: 20 µL), stirred for 12 h, centrifuged (12,000 rpm, 20 min) to collect the precipitate, washed twice with water, and resuspended M@C according to the injection volume of 500 µL for one rat; after establishing the animal model of SCI, M@C was injected into secondary SCI rats by way of tail vein, and analyzed by small animal live imager for M- PDA distribution in secondary SCI rats and its targeting capability at the center of SCI. The imaging images of secondary SCI rats were performed at 0 h, 4 h, 10 h, 24 and 36 h, and they were surgically treated in turn. Their hearts, livers, spleens, lungs, kidneys, spinal cords and brains were collected and photographed to analyze the distribution of M-PDA in each isolated tissue of the rats. To further verify the enrichment of M@C in the spinal cord, paraffin sections of spinal cord tissues were made according to HE staining method. After paraffin embedding, samples were cut longitudinally into Sect. (8 μm in thickness), and images were observed and acquired under a Confocal laser scanning microscope (CLSM Zeiss).

#### Behavioural analysis

The rats were randomly divided into 5 groups of 8 rats each. M@8G (500 µL, 10 mg/mL), 8G (500 µL, 5 mg/mL), M-PDA (500 µL, 10 mg/mL), and Saline (500 µL) were injected into the tail vein for 14 consecutive days after SCI. Healthy rats without any treatment were used as controls. The Basso Beattie Bresnahan (BBB) motor score was used to evaluate hindlimb motor function [44]. At predetermined time points (1 d, 3 d, 7 d, 14 d, 21 d, 28 d, 35 d, 42 d, 49 d, 56 d after injury), two independent members were tested and their scores were determined until both members agreed.

#### Magnetic resonance imaging (MRI) and diffusion tensor imaging (DTI) detection

At 14 days post-injury, 3.0 T MRI scanner (Discovery MR750, GE Healthcare, USA) was used to obtain all MRI data. SCI rats were anesthetized with pentobarbital sodium and placed prostrate in an 8 pathway wrist array coil (25 cm in length, 10 cm in width, 8 cm in height) for scanning, with the damaged area placed in the middle of the receiving coil. The sequence parameters of T2WI are as follows: TR = 3000 ms, TE = 77 ms, layer thickness = 1.5 mm, matrix 256 × 256, field of view (FOV): 10 × 10 mm, the number of acquisitions was 4 times. DWI sequence: TR = 2000 ms, TE = 77.5 ms, layer thickness = 1.5 mm, matrix 128 × 96, FOV = 10 × 10 mm, the number of acquisitions was 10 times; b-value = 600 s/mm^2^, DWI direction of the sagittal position was A/P. DTI sequence: TR = 5000 ms, TE minimum, layer thickness = 1.5 mm, matrix 128 × 128, FOV = 12 × 12 mm, the number of acquisitions was 1 times; b-value = 1000 s/mm^2^, DTI direction in the horizontal axis was R/L. After T2WI and DWI were obtained, the processed data was transmitted to a workstation (Advantage Windows, version 4.5; GE Healthcare, USA) to gain DTI data. Diff Tensorl Fiber Track from Functool in the workstation was used to preprocess DTI data. Tractography pathways were reconstructed utilizing DTI model based on ROI algorithm, and FA values with T10 segments were estimated.

#### GSH, MDA and SOD detection

The generation of MDA, GSH, and SOD was detected in tissue suspension using the kits as instructed by the manufacturer. The absorbance at a wavelength of 540 nm was determined using the microplate reader.

#### Western blot

Injured spinal cord epicenters 0.5 cm in size, or the CSF from patients, were ground in lysis buffer containing 50 mM Tris-HCl, 150 mM NaCl, 5 mM EDTA (pH = 7.4), 0.5% Triton-X100, and protease inhibitors. Tissue suspension was spun at 13,000 rpm at 4℃ for 10 min and stored at -20℃. Protein quantification was carried out to determine the protein concentration of the tissue suspension. 30–50 mg total protein was mixed with 5x loading buffer and heated at 100℃ for 10 min. Samples were then analyzed by using sodium dodecyl sulfate polyacrylamide gel electrophoresis (SDS-PAGE) with 8% or 10% gel for electroblotting polyvinylidene fluoride membranes. Membranes were blocked by Tris-buffered saline containing 0.2% Tween-20 (TBST) in 5% skim milk for 2 h and then incubated overnight at 4℃ with the following primary antibodies against GPX4 (1: 1000), ALOX15 (1: 1000), 4HNE (1: 1000) in TBST. Membranes were washed with TBST and incubated with horseradish peroxidase conjugated secondary antibody. Proteins were visualized using the enhanced chemiluminescence (ECL) detection system.

#### Histological analysis and immunohistochemistry

#####  Histological analysis

On day 56 post-trauma, SCI rats were anaesthetized and fixed via left ventricular perfusion with 0.9% NaCl 200mL and 4% paraformaldehyde 200 mL. Spinal cord tissue (2 cm, centered on the injury site) was taken, soaked in 4% paraformaldehyde for 24 h, dewatered and embedded in wax, and cut into Sect. (4 μm in thickness). The cut paraffin sections were dewaxed and stained with hematoxylin-eosin (HE). Imaging was performed using a scanning microscope (Precipoint M8, Germany).

##### Immunofluorescence staining

Paraffin sections were dewaxed and placed in a buffer filled with citric acid antigen repair buffer (pH = 6.0) for antigen repair. Sections were slightly dried and incubated with a histochemical pen by drawing circles around the tissue and adding drops of BSA blocking solution inside the circles for 30 min at room temperature. Sections were incubated with primary antibodies (anti-NF200: bs-10680r, anti-MBP: GB11226, Servicebio, China) respectively and kept flat overnight in a wet chamber at 4℃. Sections were washed with PBS for 3 × 5 min and then incubated with secondary antibodies for 50 min at room temperature in the dark. Subsequently, sections were washed with autofluorescent bursting agent for 5 min and rinsed with tap water for 10 min before staining with DAPI stain (G1012, Servicebio, China) to label the nuclei. Slices were sealed with anti-fluorescence quenching sealer (G1401, Servicebio, China). Images were observed and acquired under a Confocal laser scanning microscope (CLSM Zeiss). The intensity of expression of NF200 or MBP positive areas in the injury centre was assessed using ImageJ to quantify the expression of NF200 or MBP.

#### Toxic reaction

Blood samples were collected on the first 14 days after injury in tubes containing lithium heparin and centrifuged at 2000 rpm at 4℃ for 10 min for biochemical analysis. Biochemical parameters such as serum alanine aminotransferase (ALT), aspartate aminotransferase (AST), alkaline phosphatase (ALP), urea nitrogen (BUN), creatinine (Cr), uric acid (UA), lactate dehydrogenase (LDH), creatine kinase (CK) and creatine kinase isoenzyme (CKMB) were measured by use of Chemray 240 automatic biochemical analyzer. Heart, liver, spleen, lung and kidney tissues were taken 56 d after injury and subjected to HE staining and microscopic imaging according to the method as described previously.

### Collection of human cerebrospinal fluid

All eligible patients signed the informed consent form. This study was approved by the Medical Ethics Committee of the First Affiliated Hospital of Jinan University (approval number: MEC[2021]084).

We reviewed patients with SCI treated at our institution from 2018 to 2020. The exclusion criteria for this study are craniocerebral trauma or other neurological disorders with significant trauma requiring invasive intervention and/or over-excitation or sedation that prevent effective neurological examination.

The inclusion criteria for patients with acute SCI (n = 3) are adult patients with an ASIA score of grade A/B, an injury segment between C3-C7, who can effectively cooperate with neurological examination and who have undergone surgical treatment within 14 days of injury. Patients with chronic cervical medullary injury (n = 3) were included as adult patients who: (1) had ASIA score of grade A/B; (2) had an injury segment between C3-C7; (3) could effectively cooperate with neurological examination; (4) had undergone conventional decompression surgery in the acute phase and had been injured for more than 2 months until the second surgery. Cerebrospinal fluid samples were obtained following the dural incision decompression procedure. Control samples from non-SCI patients (n = 3) were obtained from patients with osteoarthritis of the knee had undergone total knee arthroplasty under combined spinal-epidural analgesia (CSEA) at the time of anesthesia.

### Statistical analysis

BBB scores were analyzed using a repeated measures ANOVA followed by a multi-factor post-comparison analysis via SPSS 26.0. In other studies, one-way ANOVA was employed for comparisons between groups. Differences were statistically significant when *p < 0.05, **p < 0.01 and ***p < 0.001.

## Result

### Preparation and characterization of M-PDA@8G nanoparticles

**Fig. 2:**
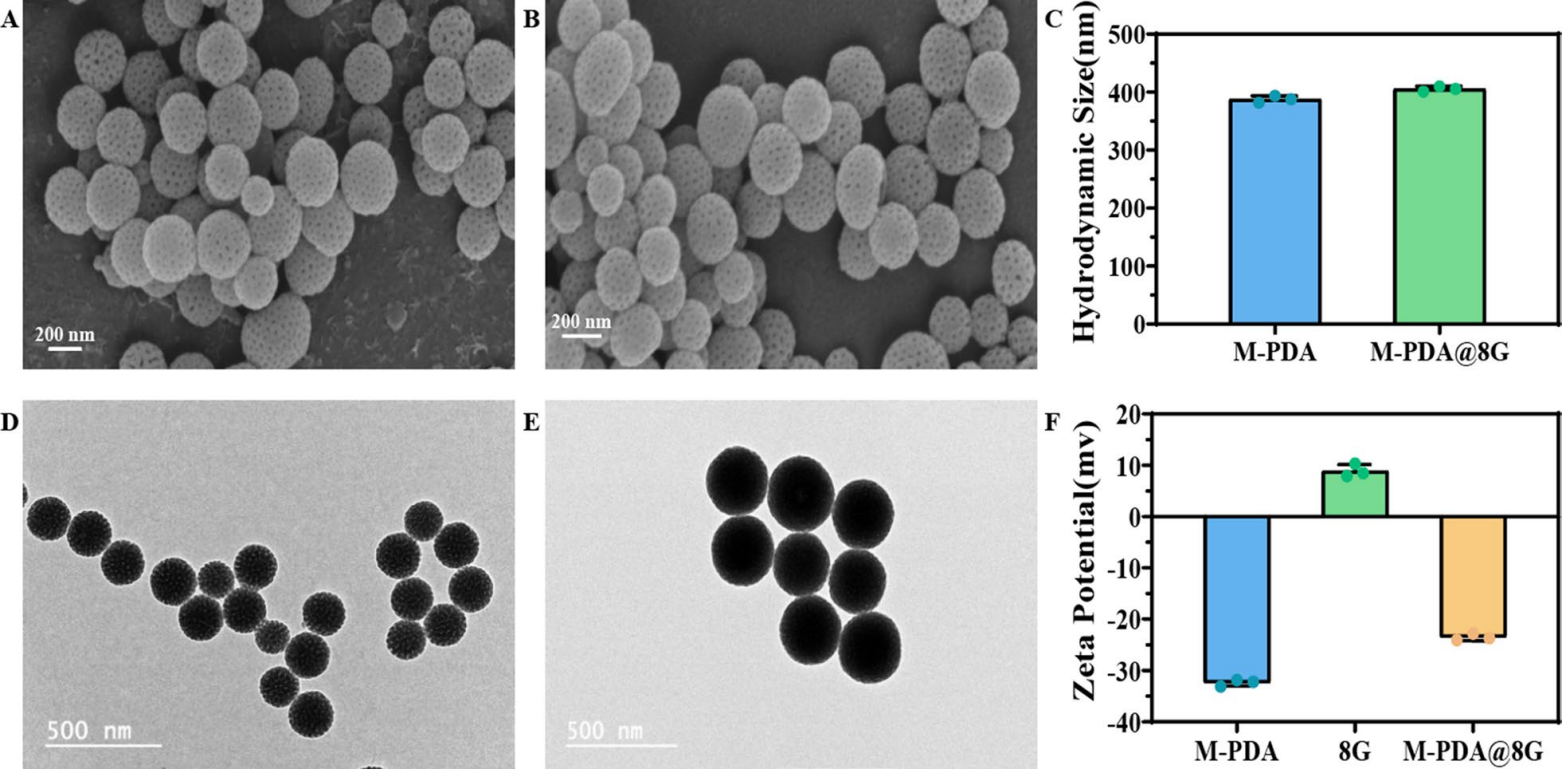
SEM image of **A** M-PDA and **B** M-PDA@8G. scale bar: 200 nm. **C** hydrodynamic size of M-PDA, M-PDA@8G. TEM images of (**D**) M-PDA and (**E**) M-PDA@8G. scale bar: 500 nm. (**F**)Zeta potentials of M-PDA,8G, M-PDA@8G

In this experiment, mesoporous M-PDA was prepared using Pluronic F-127 and 1,3,5-trimethylbenzene (TMB) as organic template molecules in aqueous solution. Ethanol and ammonia solutions were used as co-solvents and catalysts for dopamine polymerization, and then self-assembled into nanoparticles on a TMB template via π-π stacking interactions, and M-PDA was obtained after removal of the template. The morphology can be observed by scanning electron microscopy (SEM) and transmission electron microscopy (TEM). The average diameter of M-PDA was about 200 nm (Fig. 2A, D). The mesoporous structure of M-PDA, i.e. high specific surface area, is expected to achieve efficient loading of 8-ginger phenol. The morphology and particle size results of the loaded M@8G are shown in Fig. 2B, E. The particle size of the material increased after loading, and the originally clearly visible cavities on the surface of the M-PDA nanoparticles became blurred and significantly reduced in number, indicating that 8G was successfully loaded into the mesoporous structure of M-PDA. The analysis of M@8G using dynamic light scattering (Fig. 2C–F, Additional file 1: Fig. S1) showed that the potential of M-PDA itself was − 32.4 ± -2.54 mV and the particle size was 387.63 ± 0.23 nm. The potential of 8G itself was 8.88 ± 0.70 mV. After loading, the potential of M@8G increased to -23.53 ± 1.84 mV, and the particle size increased to 405.4 ± 0.28 nm. Both the potential and particle size of M-PDA changed significantly before and after 8G loading, which also further illustrates the successful preparation of M@8G. In addition, the negative surface charge of M@8G also contributes to the stability of the nanoparticles in the blood, avoiding interactions with proteins and other substances in the blood. Since positively charged nanoparticles can bind to negatively charged blood vessels and be rapidly removed from the circulation, negatively charged nanoparticles will be filtered due to charge selectivity. Therefore, the blood stability and long circulation of the nanoparticles in vivo can be enhanced, and then efficient delivery of 8G can be achieved.

**Fig. 3:**
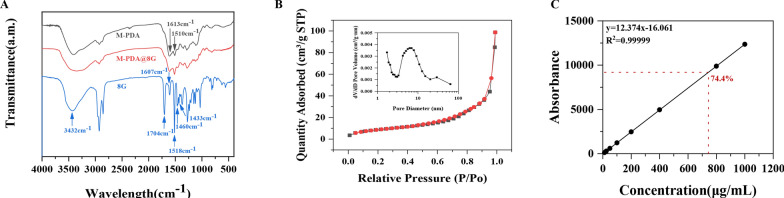
**A** Infrared spectra of M-PDA, M@8G, 8G. **B** Specific surface area analysis and pore size distribution of M-PDA. **C** Drug loading rate of M-PDA loaded with 8G

The analysis of the components of M-PDA before and after drug loading by infrared spectroscopy (Fig. 3A) shows that M-PDA has two characteristic absorption peaks at 1613 cm^− 1^ and 1510 cm^− 1^, respectively found in the N-H bending vibration in dopamine and the superposition of the C = C backbone and N-H shear vibration in the benzene ring [41]; 8G has a stretching vibration peak at 3432 cm^− 1^ for O-H, a characteristic absorption peak at 1704 for C = O, and a characteristic absorption peak at 1518 cm^− 1^, 1607 cm^− 1^, 1460 cm^− 1^, and 1433 cm^− 1^ for the benzene ring [45]. In the spectrum of M-PDA@8G after drug loading, we found that the number and intensity of the characteristic absorption peaks of 8G both significantly reduced; the characteristic absorption peaks in the benzene ring, in particular, were basically masked by the IR spectrum of M-PDA, which indicated that the two interacted, further suggesting the successful preparation of M-PDA@8G. Brunauer-Emmett-Teller (BET) analysis (Fig. 3B) showed that the N_2_ adsorption-desorption curve of M-PDA matched the type IV isotherm of the mesoporous material, and the specific surface area of M-PDA was, by calculation, about 30.72 m^2^/g with a pore size of about 8.19 nm. The peak at 29.45 nm in the adsorption curve was attributed to the pore structure caused by the removal of the template. Further analyses of the supernatant of M@8G after drug loading were made using high performance liquid chromatography (Fig. 3C), and the results showed that the drug loading rate of 8G in M@8G was as high as 74.4%, displaying the advantages of the mesoporous structure in terms of drug loading. Drug release is at around 39% (Additional file 1: Fig. S2).

### In vitro anti-LPO studies

**Fig. 4:**
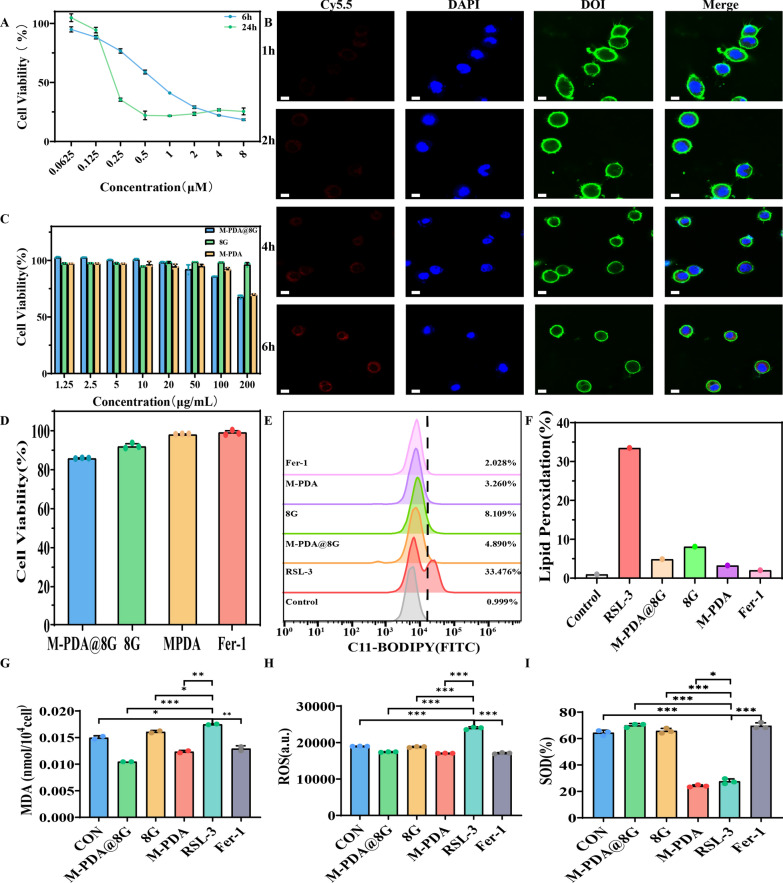
**A** Plots of HT22 cell numbers versus different concentrations of RSL-3 incubated for 6 and 24 h. **B** Confocal images of HT22 cells after co-incubation with M@8G for 1, 2, 4 and 6 h (Scale bar = 10 μm). **C** HT22 cells viability evaluation after incubating with M-PDA, 8G, M@8G. **D** HT22 cells viability evaluation after incubating with M-PDA, 8G, M@8G, Fer-1. **E** Comparison of the anti-LPO activity of M-PDA, 8G, M@8G, and Fer-1. **F** Quantitative analysis of the anti-LPO capacity of M-PDA, 8G, M@8G, and Fer-1. (**G**), (**H**), (**I**) Intracellular MDA, ROS, SOD levels in HT22 cells after induction treatment with RSL-3 and treatment with M-PDA, 8G, M@8G, Fer-1

RSL3 is the inhibitor of the glutathione peroxidase 4, which can inhibit the cysteine/glutamate amino acid transporter system xc-, blocks GSH synthesis and promotes iron death. Through experimental validation, we found that the cell survival rate of HT22 cells was roughly 40% when co-incubated with 1µM RSL-3 for 6 h (Fig. 4A). We used laser confocal scanning microscopy to analyze the endocytosis of M@8G by HT22 cells. Figure 4B shows the confocal images of the M@8G nano-delivery platform after co-incubation with HT22 cells for 1 h, 2 h, 4 and 6 h. We labelled M@8G, nucleus and cell membrane as red, blue and green fluorescence respectively, and judged the endocytosis effect of HT22 cells on M@8G based on the intensity of fluorescence. As can be seen from the graph, the red fluorescence became increasingly stronger over time, and the purple part became more and more obvious after superimposition, indicating that a larger number of M@8G nanoparticles entered HT22 cells, which reached the maximum value at 6 h. The cytotoxicity with different concentrations of M-PDA, 8G and M@8G in HT22 cells was then investigated separately. As shown in Fig. 4C, HT22 cells had a high cell survival rate after co-incubation with each concentration of M-PDA, 8G and M@8G for 24 h, but when the concentration of M@8G reached 200 µg/mL, it would have certain toxic side effects on the cells and the cell survival rate was only approximately 70%. Therefore, through cytotoxicity experiments, we finally selected a concentration of M@8G at 100 µg/mL, which resulted in a cell survival rate of over 85%. Subsequently, we incubated HT22 cells with the selected concentrations of M-PDA, 8G, M@8G and Fer-1(Ferrostatin-1 is a commonly used and potent ferroptosis inhibitor) as a control for 24 h and found that the cell survival rate of all groups was above 85% (Fig. 4D), indicating that our material had no significant toxic effects on the cells.

ROS-mediated LPO is a key step in driving ferroptosis [9, 46]. High intracellular levels of reduced glutathione (GSH) and superoxide dismutase (SOD) can downregulate ROS production levels [9]. The end products of LPO are reactive aldehydes such as malondialdehyde (MDA) and 4-hydroxynonenal (4HNE), highly reactive aldehydes that can further impair protein/DNA/membrane function [47, 48]. To investigate the anti-LPO effect of nanoparticles, HT22 cells were treated with M-PDA, 8G, M@8G, and Fer-1 respectively after RSL-3 induction treatment. Experimental results showed that SOD expression levels were significantly higher in the 8G and M@8G treatment groups (Fig. 4I). Among them, the M@8G group had the best treatment effect, which indicated that the anti-LPO ability was significantly enhanced by the combination of M-PDA and 8G [49].

### Ferroptosis factor assay in human cerebrospinal fluid

**Fig. 5:**
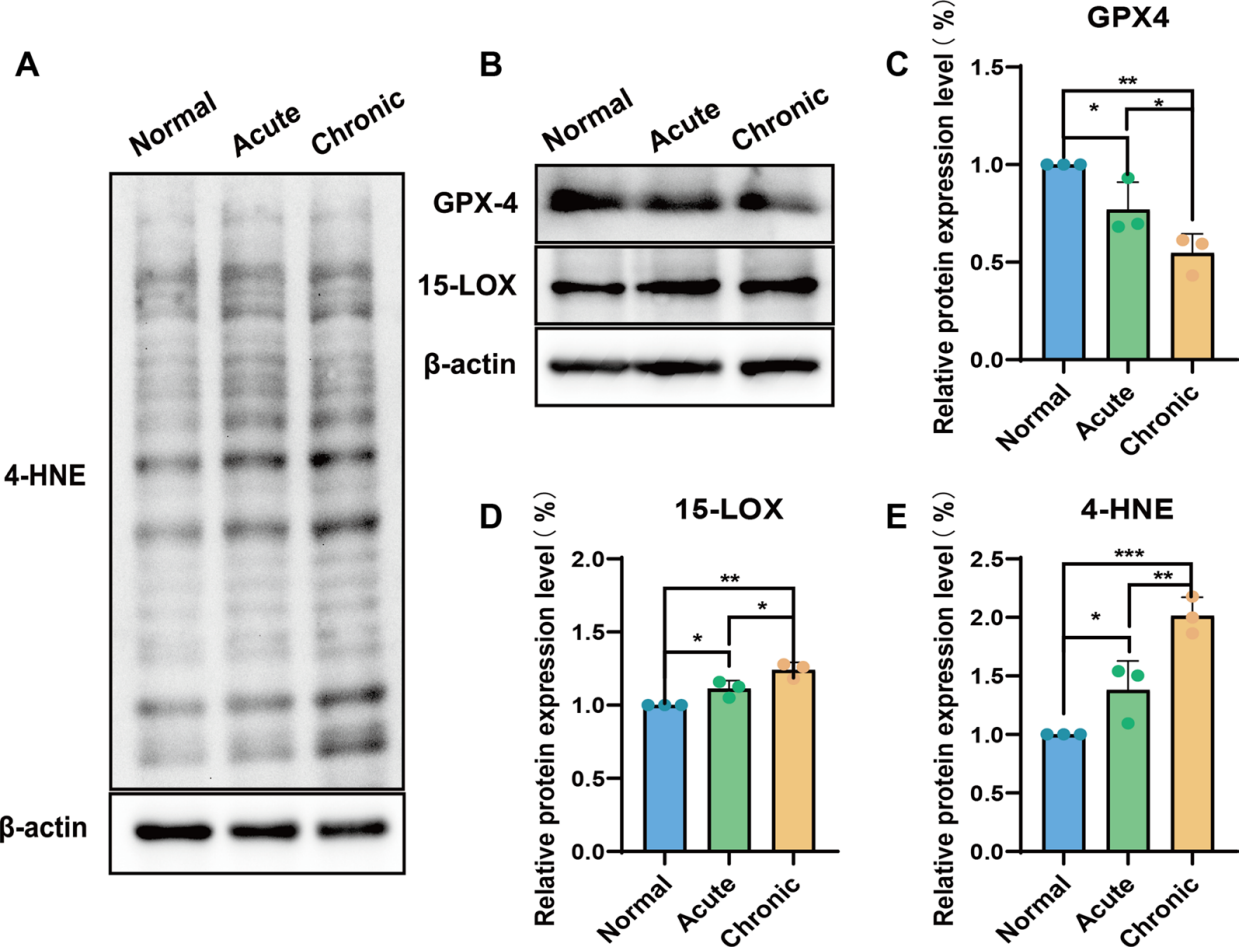
Detection of ferroptosis in human cerebrospinal fluid: (**A**, **B**) Western blotting detected the levels of GPX4, 15-LOX, 4-HNE factors in cerebrospinal fluid of acute and chronic SCI patients and non-SCI patients. (**C**-**E**) Quantitative analysis of the indicators imaged in (**A**, **B**) (n = 3)

Studies have shown that the expression levels of certain biomarkers in the cerebrospinal fluid can be used as an important tool for grading SCI [50–53]. We examined the expression levels of ferroptosis factor in the cerebrospinal fluid of acute, chronic and non-SCI patients. Compared to non-SCI patients, SCI patients had reduced levels of GPX4 and increased production of 15-LOX/4-HNE in the cerebrospinal fluid, both of which, from a different perspective, indicated that ferroptosis occurred in SCI patients (Fig. 5). At the same time, ferroptosis progressed even after the patient received clinical treatment in the acute phase of SCI, suggesting that inhibition of ferroptosis may be one of the effective ways to treat SCI in the long term. In our study, M@8G micelles showed good inhibition of ferroptosis, signifying that M@8G may be an effective way to treat SCI in the long term. In addition, studies have shown that 8G has the ability to inhibit cellular inflammation, autophagy and apoptosis, indicating that M@8G nanoparticles have promising applications in the treatment of SCI.

### In vivo targeting ability of M-PDA

**Fig. 6:**
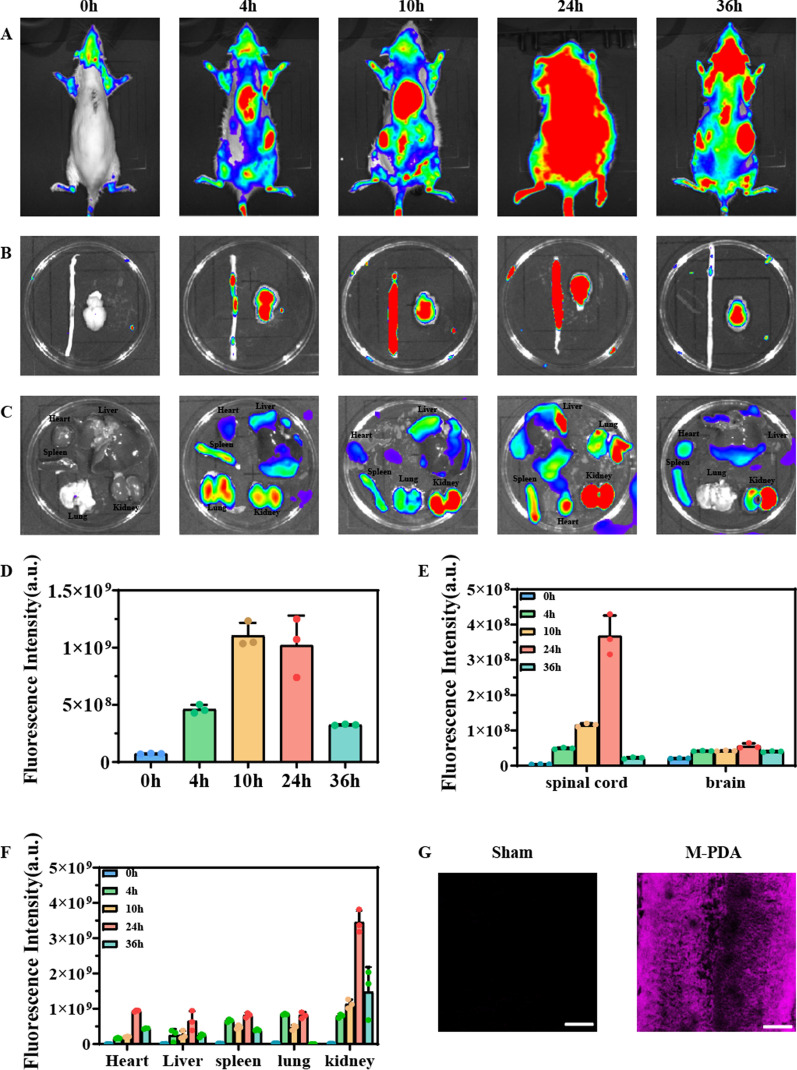
**A** In vivo fluorescence imaging of rats 36 h after Cy5.5-labelled M-PDA injection via tail vein. **B** In vitro fluorescence imaging of M-PDA in the spinal cord and brain. **C** In vitro fluorescence imaging of M-PDA in the major organs of the rat. **D** Quantification of the fluorescence signal intensity of M-PDA at the site of SCI. **E** Quantification of the fluorescence signal intensity of M-PDA in the isolated SCI center and brain of rats. **F** Quantification of the fluorescence signal intensity of M-PDA in major organs of rats. **G** Fluorescence images of spinal cord tissue in SCI rats after tail vein injection of Cy5.5-labelled M-PDA (Scale bar = 200 μm)

In order to evaluate the in vivo targeting ability of M-PDA, we labelled M-PDA with fluorescent dye Cy5.5 and verified it by a small animal imager. SCI rats were injected with M-PDA loaded with Cy5.5 after surgery. By comparing in vivo and in vitro tissues at 0 h, 4 h, 10 h, 24 and 36 h, we found (Fig. 6A, B, C) that the fluorescence signal at the site of SCI gradually increased, reached the maximum value at 24 h after injection, and then began to decay. Through quantitative analysis of fluorescence signal intensity of living and in vitro tissues (Fig. 6D, E, F), we can more intuitively see the enrichment effect of M-PDA in SCI. Amazingly, we found that M-PDA was enriched not only in the SCI site, but also in the brain, which preliminarily revealed that M-PDA could penetrate the blood-brain barrier and blood-spinal cord barrier after SCI. Through the analysis of immunofluorescence sections (Fig. 6G), we found that CY5.5-labelled M-PDA would gradually accumulate at the site of SCI over time, further demonstrating that M-PDA could penetrate the blood-brain barrier and blood-spinal barrier to reach the site of SCI after SCI.

### Inhibitory effect of M@8G on secondary SCI

#### Behavioral analysis

**Fig. 7:**
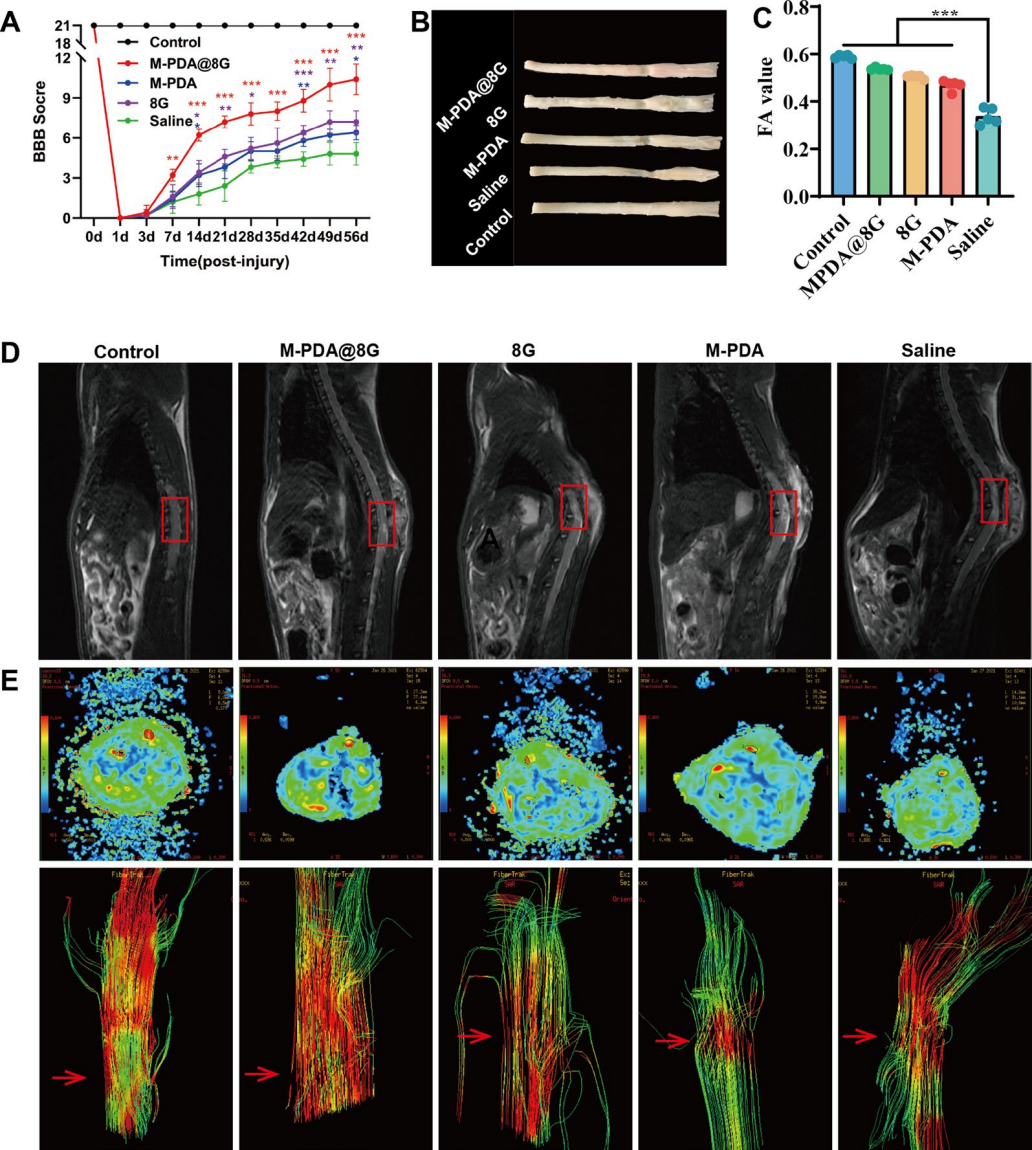
Assessment of motor function recovery. **A** BBB scores of rats in each experimental group at different times (n = 5). **B **Images of the appearance of the spinal cord in various groups 56 days after injury. **C** FA values obtained by DTI in the injury area of the different treatment groups at day 14 post-injury (n = 5). **D** T2-weighted MRI images of rats in different experimental groups on day 14 after injury (red oval indicates the center of injury). **E** FA images (upper panel) and DTI images (lower panel, red arrow indicates the site of injury) of rats in different experimental groups on day 14 after injury

To assess the long-term efficacy of different treatments, normal rats were used as a positive control group. On a daily basis, SCI rats were injected once with M-PDA@8G micelles, 8G, PDA or saline via tail vein for 14 days. Behavioral analysis of different experimental groups was performed using Basso, Beattie and Bresnahan grading scales (BBB scores) during 56 days of injury. The evolution of the scores for all groups is shown in Fig. 7A. After SCI, the rats had a complete loss of motor function in the hind limbs, but the BBB score gradually became higher over time. Compared to the Saline group, the loaded M@8G, 8G, and PDA groups all showed some improvement, to a certain extent. According to the previous section, PDA carriers have good targeting ability for SCI and other effects such as antioxidant ability. Although not loaded with 8G, the PDA group also had therapeutic effects. On the other hand, the M@8G experimental group scored significantly higher than the other experimental groups, which may be due to the combined effect of PDA and 8G. The spinal cords of the rats in different experimental groups were sampled (Fig. 7B), and the comparison showed that the spinal cord of the M@8G-treated group was, relatively speaking, structurally intact. These results suggested that M@8G had the optimal therapeutic effect on motor function.

#### Magnetic resonance and diffusion function imaging

In order to monitor the early treatment effect and evaluate the prognosis of different experimental groups, the lesion site was directly observed by 3.0 T MRI-T2 phase and diffusion tensor imaging (DTI) technology on the 14th day after injury (Fig. 7D, E). In acute SCI, MRI and T2-weighted images show ill-defined hyperintensity, reflecting spinal cord edema and hemorrhage at the trauma site [54]. Compared with the other treatment groups, the area of hyperintensity in the saline group was wider; the hyperintensity area in the M@8G group mainly concentrated in the dorsal region of the spinal cord rather than in the full thickness, with fewer segments involved. As a methodology for quantitatively studying moisture diffusion, DTI provides information on the directionality of diffusion, which can reveal the orientation of fibers. The fractional anisotropy (FA) obtained from DTI can reflect the degree of diffusion anisotropy. FA values close to 1 indicate regular and directional tissue [55, 56]. According to the DTI results 14 days after trauma, the nerve fibers in the control group and the M@8G treatment group had tight and regular connections, while the fiber connections in the other treatment groups were broken. Compared with other experimental groups, the FA value in the SCI center of the M@8G treatment group on the 14th day after SCI was closer to that of the control group. Imaging detection showed that M@8G micelles had better control effect on secondary SCI (Fig. 7C).

#### Histopathological observations

**Fig. 8:**
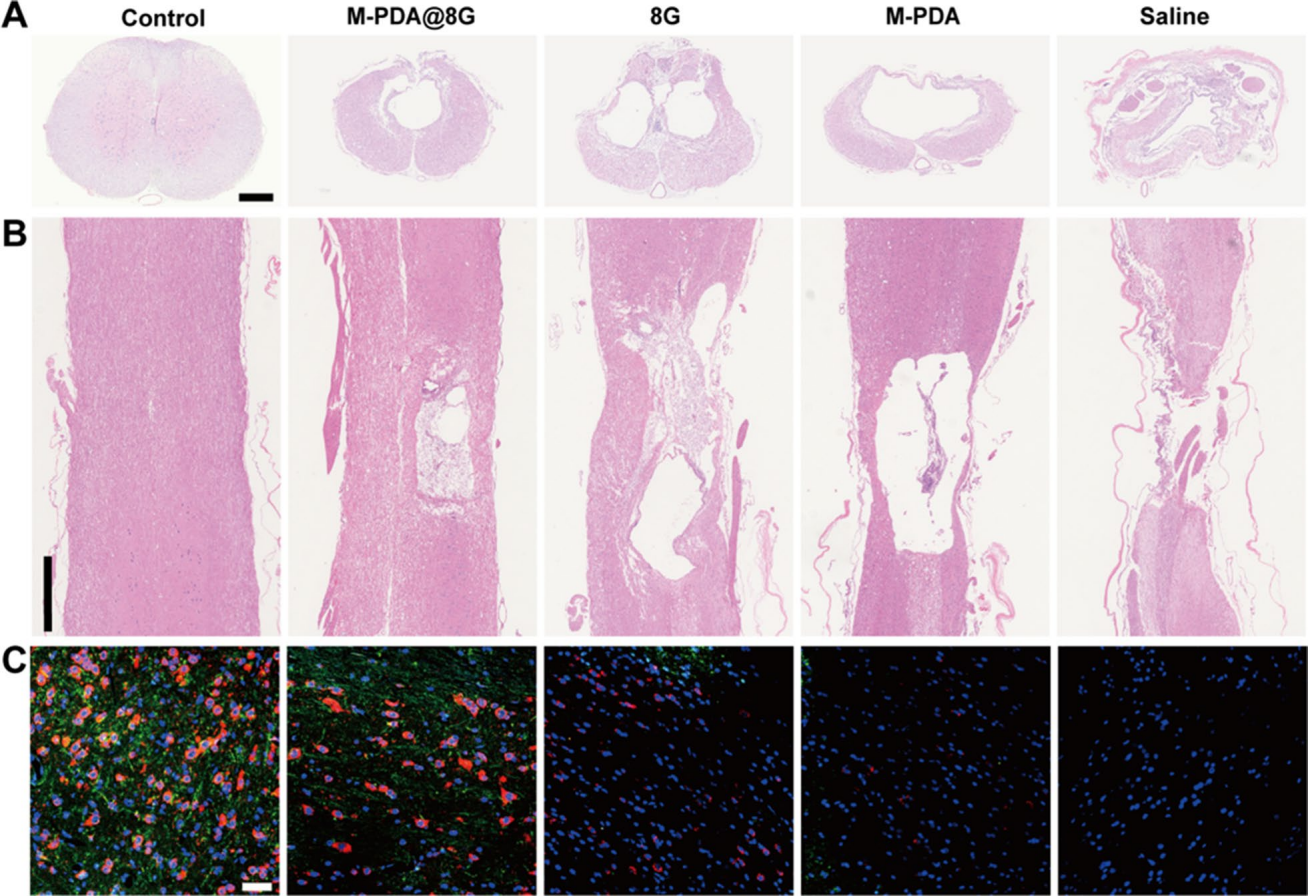
Histopathological observations. **A** H&E staining of spinal cord cross-sections in each treatment group on day 56 after SCI (Scale bar = 500 μm). **B** H&E staining of the sagittal plane of the spinal cord in each treatment group on day 56 (Scale bar = 1 mm) **C** Day 56 after injury, MBP (green, myelin marker) and NF200 (red, axis markers) were used respectively immunofluorescence staining of spinal cord sections (Scale bar = 50 μm)

To observe pathological changes at the site of injury, hematoxylin-eosin (H&E) staining (Fig. 8A, B) were performed on the spinal cord in each group on day 56 post-trauma. As with the appearance of the spinal cord on day 56, the Saline group had the least integrity and continuity of the spinal cord, with complete loss of spinal cord morphology. The groups treated with M@8G, PDA and 8G all showed varying degrees of improvement in spinal cord integrity. By comparison, the M@8G lesion cavity was the smallest, suggesting that M@8G had the best therapeutic effect.

**Fig. 9:**
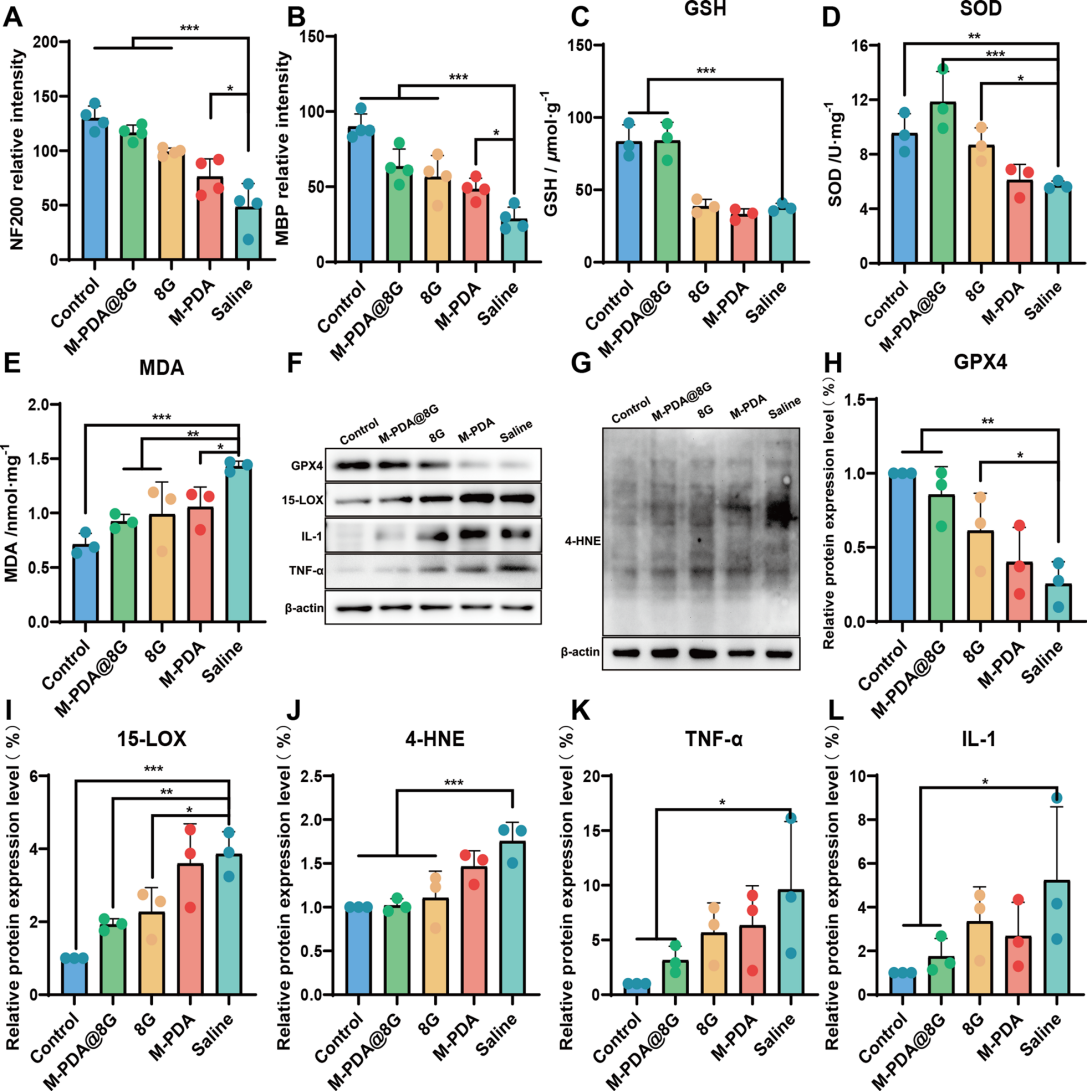
Pharmacological effects and statistical analysis of M-PDA@8G in the treatment of SCI (**A**, **B**) Quantification of NF200 and MBP in immunofluorescence sections of spinal cord by ImageJ (n = 4). **C**, **E** Levels of GSH, SOD, and MDA in the spinal cord of different experimental groups on the 14th day after injury (n = 3). **F, G** Effects of different treatments on GPX4, 15-LOX-2, 4-HNE, IL-1β, and TNF-α levels on day 14 post-injury detected by Western blotting. (H-L). Quantitative analysis of the indicators imaged in **F**, **G** (n = 3)

After SCI, axonal demyelination peaks on day 1 and decreases between 7 and 14 days after injury, followed by remyelination [57, 58]. Studies have shown that intervening in axonal demyelination and remyelination is one of the most important ways to treat spinal cord injuries [58, 59]. To evaluate the effect of different treatments on axonal demyelination and myelin regeneration in the injured center after SCI, we performed immunofluorescence staining of axonal myelin with MBP and NF200 antibodies (Fig. 8C). Subsequent quantification of NF200 and MBP expressed in immunofluorescence sections of the spinal cord was performed using Image J (Fig. 9A, B). Confocal imaging in the Saline group showed that MBP and NF200 were barely expressed in the injury area, indicating a significant loss of myelin and axons at the lesion site. The 8G and M-PDA groups retained part of myelin and axons in the injury center, but the number of myelin and axons was significantly less than that in the M@8G group. This may be attributed to the targeted distribution and combined protective effect of M-PDA and 8G on nerve cells.

#### Pharmacological effects

To investigate the molecular mechanism of ferroptosis after SCI, spinal cord tissue was taken for testing on day 14 after treatment (Fig. 9E–J). Compared to the Saline group, down-regulation of 4-HNE/MDA expression was found in the M-PDA group, which demonstrated the anti-LPO function of M-PDA. Compared with the Saline group, GPX4 expression was upregulated in the M@8G and 8G groups;15-LOX and 4-HNE/MDA expressions were downregulated, indicating that M@8G and 8G treatments had the anti-ferroptosis function. Thanks to the targeting ability and anti-LPO of PDA, the optimal ability to inhibit ferroptosis was found in the M@8G group. In contrast, the targeting of PDA enhanced the ability of 8G to inhibit ferroptosis. As one of the antioxidant systems that eliminate reactive oxygen species in vivo, GSH is involved in anti-ferroptosis by regulating GPX4 activity [60, 61]. Overexpression of SOD can prevent LPO in vivo [62]. As can be seen from the statistics, expression levels of GSH and SOD were similar in control and M@8G treatment groups (Fig. 9C, D). LPO can also play an important role in many inflammatory diseases, frequently mediating pro-inflammatory changes [63, 64]. The inflammatory response is an important aspect of secondary SCI and severely impedes neuronal and axonal regeneration [65]. In the present study, IL-1β and TNF-α levels were lower in the rats treated with M@8G than those in the rats from the other groups (Fig. 9K, L), indicating reduced local inflammation.

#### Assessment of adverse reactions

**Fig. 10:**
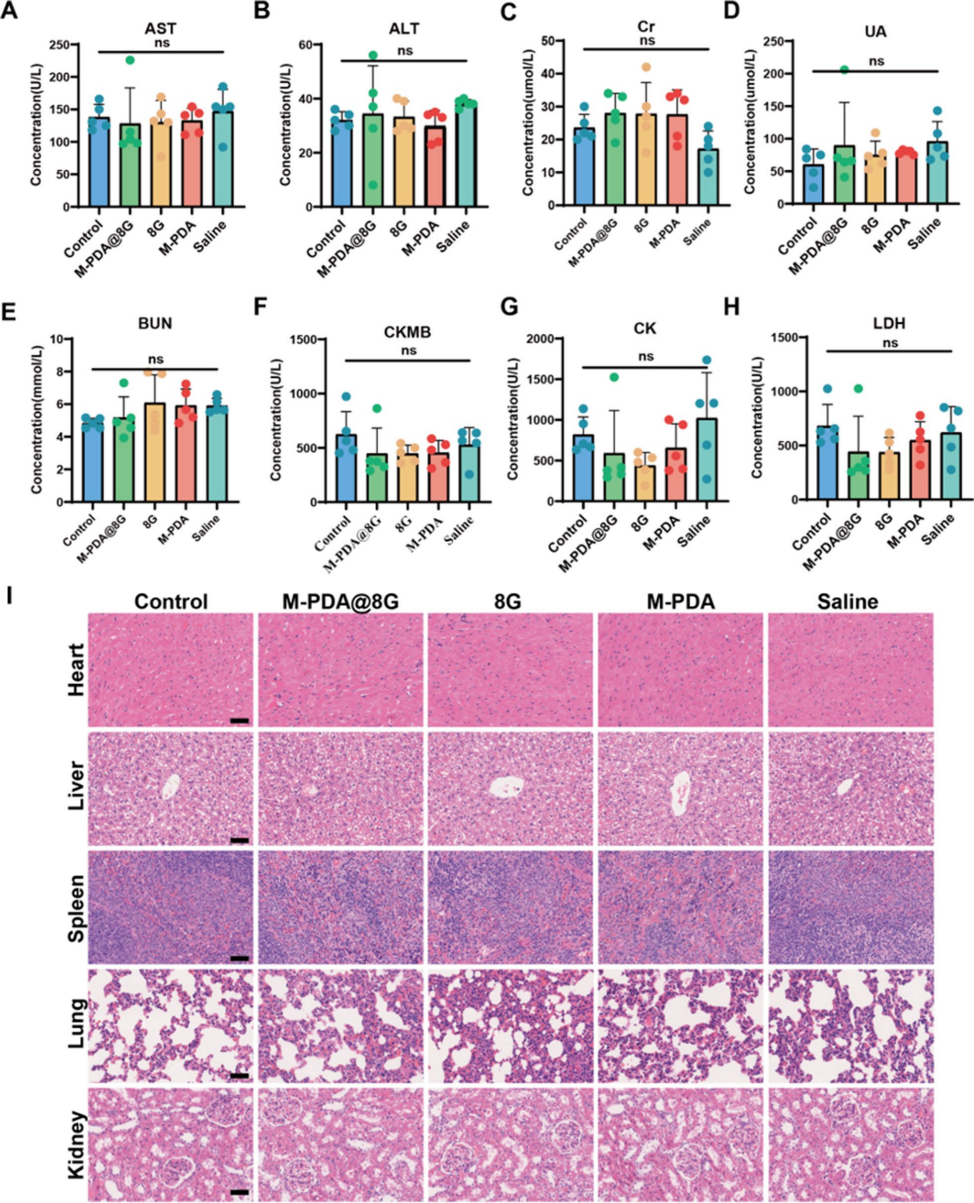
Assessment of adverse reactions: **A**-**H** levels of BUN, Cr, UA, ALT, AST, LDH, CK, and CKMB in different treatment groups after 14 days of drug treatment. **I** H&E staining was performed on the heart, liver, spleen, lung and kidney in different treatment groups after 56 days of drug treatment (Scale bar = 100 μm)

To evaluate whether M@8G micelles are potentially toxic during treatment, we performed hematological tests on the 14th day after trauma, including BUN, Cr, UA, ALT, AST, LDH, CK, CKMB measurements. We found no significant differences in these metrics between the control and other treatment groups (Fig. 10A–H). In addition, we performed H&E staining on the tissues (heart, liver, spleen, lung and kidney) obtained at 56 day (Fig. 10I), and there was no obvious sign of damage in the tissues of each experimental group, indicating the biocompatibility of M@8G micelles.

## Discussion

SCI often results in permanent functional impairment, including sensory, motor and autonomic nerve defects, leaving most patients incapacitated for work and life [66–68]. Secondary SCI, which is the body’s secondary response to the primary injury, occurs minutes after the trauma and can last for a few days or weeks. The mechanisms of secondary SCI are complex, but the prognosis of SCI can be improved if human intervention is involved during this phase.

Many mechanisms are involved in the secondary cascade of injury following SCI. However, most currently adopted therapeutic strategies target only one or a few elements of the injury cascade and have been largely unsuccessful in clinical trials [69].8G, one of the main functional substances of ginger, has been shown to have inhibitory properties on inflammation, antioxidant, autophagy and apoptosis [10–14]. However, the disadvantages of 8G, such as low water solubility, poor chemical stability and rapid metabolism, have limited its application in SCI. For this reason, we constructed the M@8G nano-delivery platform [70, 71]. In this study, we have demonstrated that M@8G has good biocompatibility, high drug loading capacity and it can cross the blood-spinal cord barrier after SCI, and then target accumulation at the site of SCI in the acidic microenvironment of SCI, which in turn dissociates and releases 8G to achieve slow drug release for more than 24 h. Meanwhile, as polydopamine has certain free radical scavenging ability, it can improve the microenvironment of SCI together with 8G, so that 8G can better exert its drug effect [72–74].

In the present study, we also found that 8G had an inhibitory effect on ferroptosis in secondary SCI. We first verified in vitro experiments that both 8G and M-PDA had good anti-LPO ability, and that the synergistic effect of M-PDA made the effect of 8G more potent. Subsequently, in vivo experiments, we confirmed the role of 8G inhibiting ferroptosis in secondary SCI. The targeting and pharmacological effects of M-PDA increased the concentration of 8G at the site of injury and improved the microenvironment of the injury center, resulting in a more pronounced inhibition of ferroptosis in the M@8G group. In addition, we confirmed that M@8G reduced the local inflammatory response of SCI.

On this basis, M@8G nanoparticles achieved better recovery of motor function in the treated SCI rat model, as reflected by higher BBB scores. Imaging tests were performed on day 14 after SCI to assess early treatment outcome and prognosis. The M@8G treatment group showed a smaller range of high signal areas on MRI-T2 compared to the other treatment groups, while DTI showed tighter and more regular nerve fiber connections. Histological analysis and immunohistochemical staining of the treatment groups at day 56 post-trauma showed relatively intact structures in the M@8G treatment group, while more distinct myelin sheaths and axons were observed in immunohistochemical staining of the injury centers. This evidence suggests that M@8G can target SCI and exert synergistic pharmacological effects to achieve effective treatment of SCI.

Finally, and more intriguingly, ferroptosis evolves in humans after SCI, which provides important data to inform our nano-delivery platform and even other ferroptosis-inhibiting therapeutic options in SCI. We examined cerebrospinal fluid samples from acute, chronic and non-SCI patients, and the results suggest that ferroptosis occurs locally to the injury in SCI patients. Moreover, ferroptosis progressed locally even after the patients were in the acute phase of SCI and underwent conventional surgical treatment. Given the superior ability of M@8G to inhibit ferroptosis, M@8G can be viewed as a long-term treatment for SCI.

In conclusion, our M@8G nano-delivery platform, featured by its sufficient biocompatibility and high drug loading capacity, can penetrate the blood-spinal cord barrier and can be targeted to accumulate at the site of SCI. Furthermore, the M@8G nano-delivery platform acts as an anti-inflammatory and anti-ferroptosis agent, providing a safe and effective strategy for the long-term clinical treatment of SCI. With many diseases currently facing similar problems to SCI, our novel nano-delivery platform holds promise for multi-dimensional applications. We also believe that this bionic and easily prepared nano-delivery platform is a trend for future clinical applications.

## Supplementary information

**Additional file 1**: **Figure S1**. DLS plot showing the particle size ofM-PDA andM-PDA@8G. **Figure S2**. Drug release curves for M@8G.

## Data Availability

The data used to support the findings of this study are available from the corresponding author upon request.

## Abbreviations

HT22: Mouse Hippocampal Neurons
SCI: spinal cord injury
BSCB: Blood-Spinal Cord Barrier
8G: 8-Gingerol
LPO: Lipid Peroxidation
CCK-8: Cell counting kit-8
CLSM: Confocal laser scanning microscope
DMEM: Dulbecco’s modified eagle’s medium
DMSO: Dimethyl sulfoxide
FBS: Fetal bovine serum
GSH: Glutathione
M-PDA: Mesoporous polydopamine
PBS: Phosphate buffer solution
ROS: Reactive oxygen species
MDA: Malondialdehyde
SOD: Superoxide dismutase
WB: Western Blot
SEM: Scanning electron microscope
TEM: Transmission electron microscope
HPLC: High Performance Liquid Chromatography
