# Supplementary material for: Mesoporous polydopamine delivering 8-gingerol for the target and synergistic treatment to the spinal cord injury
Source: J Nanobiotechnology. 2023 Jun 14;21:192. doi: 10.1186/s12951-023-01896-1 (PMC10268369; doi:10.1186/s12951-023-01896-1)
Supplement: Supplementary file 1 — Additional file 1: Figure S1. DLS plot showing the particle size ofM-PDA andM-PDA@8G. Figure S2. Drug release curves for M@8G. [file 12951_2023_1896_MOESM1_ESM.pdf]

Mesoporous polydopamine delivering 8-Gingerol for the target and synergistic treatment to the spinal cord injury

Jinpei Yang[a, b, c], Meng Wang[d], Shuai Zheng[a], Ruodong Huang[d], Ganjun Wen[c], Pan Zhou [a], Wenbo Wang[d], Shihao Zhou[d], Xinlin Jiang[d], Shuangjiang Liu[a], Zhizhong Li[a, f, *], Dong Ma[d, e, *], Genlong Jiao[a, c, *]

[a] Department of Orthopaedics, the First Affiliated Hospital of Jinan University, Jinan University, 613 Huangpu Avenue West Road, Guangzhou 510630, Guangdong, China

[b] Department of Orthopaedics, Huizhou Third People's Hospital, Guangzhou Medical University, Huizhou, 516002, Guangdong, China

[c] The Sixth Affiliated Hospital of Jinan University, Jinan University, Dongguan 523573, Guangdong, China

[d] Key Laboratory of Biomaterials of Guangdong Higher Education Institutes, Engineering Technology Research Center of Drug Carrier of Guangdong, Department of Biomedical Engineering Jinan University Guangzhou, 510632, China

[e] MOE Key Laboratory of Tumor Molecular Biology, Jinan University, Guangzhou, 510632, China

[f] The Fifth Affiliated Hospital of Jinan University, Jinan University, Heyuan 51700, Guangdong, China

*Corresponding author.

E-mail address: lizhizhongjd@163.com (Zhizhong Li), tmadong@jnu.edu.cn (Dong Ma), jiaogenlong@163.com, (Genlong Jiao).

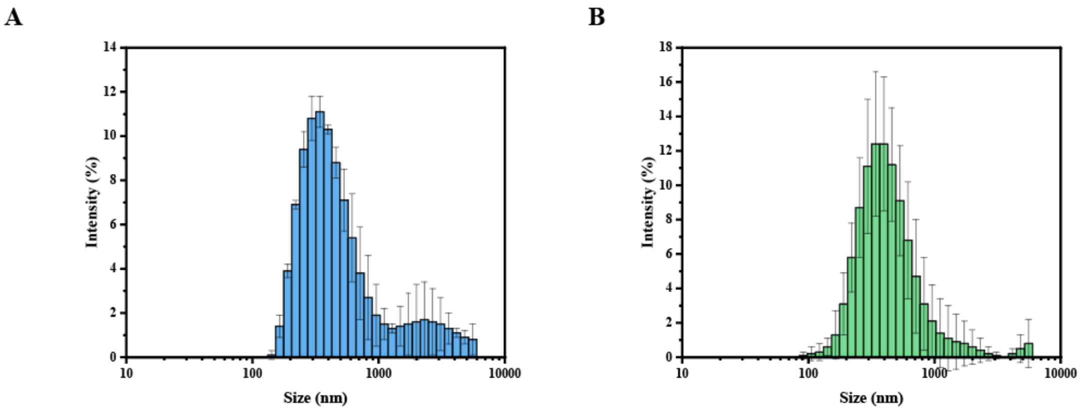

Fig.S1. DLS plot showing the particle size of (A) M-PDA and (B) M-PDA@8G.

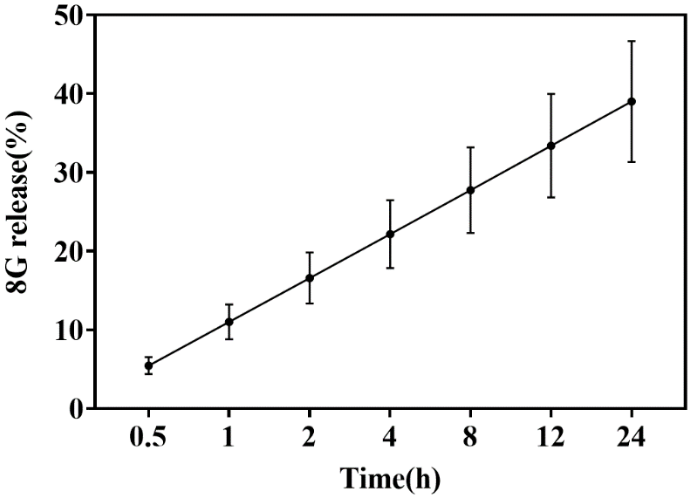

Fig.S2. Drug release curves for M@8G.
